# Editorial: Molecular biomarkers of cardiometabolic disease

**DOI:** 10.3389/fendo.2024.1471571

**Published:** 2024-08-12

**Authors:** Mirjana Macvanin, Aleksandra Klisic, Esma R. Isenovic

**Affiliations:** ^1^ Independent Researcher, Belgrade, Serbia; ^2^ Center for Laboratory Diagnostic, Primary Health Care Center, University of Montenegro-Faculty of Medicine, Podgorica, Montenegro; ^3^ Department of Radiobiology and Molecular Genetics, VINČA Institute of Nuclear Sciences - National Institute of the Republic of Serbia, University of Belgrade, Belgrade, Serbia

**Keywords:** biomarker, cardiometabolic disease, type 2 diabetes, cardiovascular disease, risk assessment, diagnostic biomarker, microRNA, machine learning

Cardiometabolic disease (CMD) is a leading global cause of mortality and refers to a complex sequence of pathophysiological processes resulting in both metabolic and cardiovascular disease (CVD). Central to its development is insulin resistance, which involves perturbed regulation of glucose levels, inflammation, and endothelial dysfunction. Timely detection, accompanied by appropriate intervention, is crucial in preventing CMD-associated complications such as type 2 diabetes mellitus (T2DM) and CVD. However, the major challenge of CMD management lies in its complex and diverse manifestations that often defy prediction based solely on traditional risk factors due to the absence of reliable and accurate molecular CMD biomarkers. The present Research Topic, “Molecular biomarkers of cardiometabolic disease” aims to showcase new developments in identifying, characterizing, and evaluating novel molecular biomarkers for CMD risk assessment, screening, and diagnosis/prognosis. By covering novel research advances and clinical trends in identifying and evaluating molecular CMD biomarkers, this Research Topic offers insights into novel potential diagnostic solutions that may support early interventions and improve outcomes.

This Research Topic has collected nine original research articles. The study by Garcia et al. reports that serum low-density lipoprotein receptor-related protein 1 (sLRP1) levels, which predict cardiovascular risk, are upregulated, whereas atrial natriuretic peptide (ANP) levels are downregulated in T2DM patients at disease onset. Increased sLRP1 and decreased ANP levels are normalized in the T2DM patients that reached optimal glycemic and metabolic control. The authors propose that the sLRP1/ANP ratio could be a reliable marker of cardiometabolic function and the cardiovascular benefits of glycemic control in T2DM patients.

Gou et al. combined experimental and bioinformatics approaches to investigate energy metabolism-related genes (EMRGs) in heart failure with preserved ejection fraction (HFpEF). Gene expression profiles in the HFpEF mouse dataset were compared to control groups to identify differentially expressed EMRGs (DE-EMRGs), and the potential biomarkers with diagnostic value were screened using machine learning algorithms. The analysis revealed five potential diagnostic biomarkers for HFpEF as well as several promising therapeutic targets that deserve future investigation.

Chen et al. conducted a cross-sectional study to assess the relationship between serum testosterone (TT) and apoB in diverse populations exposed to different factors. The study reports a statistically significant, inverse correlation between serum TT concentration and apoB concentration. The authors suggest that an investigation of the correlation between serum TT and apoB may be used for screening individuals with CVD risk within the population that exhibits normal or low LDL-C levels.

A retrospective observational study by Bosco et al. investigated SLCO1B1 rs4149056 impact on LDL-C target achievement after lipid-lowering therapy optimization in men and women with familial hypercholesterolemia (FH). The authors found that the genotype effect of SLCO1B1 rs4149056 is more pronounced in FH women since the prevalence of subjects on the LDL-C target and high-intensity lipid-lowering therapy was significantly lower in FH women with SLCO1B1 rs4149056 than in other groups.

A study by Chen et al. reports that the serum level of fetuin-A, which is a glycoprotein that acts as an inhibitor of insulin secretion and arterial calcification progression (1), negatively correlates with the risk of TTAs and is accompanied by decreased descending thoracic aortic diameter. These findings suggest that monitoring of serum fetuin-A levels may be used in the early detection and diagnosis of TTA.

Su et al. performed a drug target Mendelian randomization (MR) analysis on seven genetic variants encoding lipid-lowering drug targets (LDLR, HMGCR, NPC1L1, PCSK9, APOB, APOC3, and LPL) to explore the impact of lipid-lowering drug targets on erectile dysfunction (ED). The results show that APOB inhibitors are associated with an increased risk of ED occurrence, whereas APOC3 inhibitors, LDLR, and LPL agonists are significantly associated with a reduced risk of ED occurrence. LDLR AND LPL agonists were also significantly associated with increased TT levels. The authors propose that APOB, APOC3, LDLR, and LPL may be new drug target candidates for ED treatment.

Xu et al. assessed clinical correlation and demographic characteristics of hyperhomocysteinemia (HHcy) within the Chinese urban population with hypertension, focusing on the identification of risk factors for HHcy in hypertensive patients. The authors report high HHcy prevalence in the Chinese urban population, as well as a significant association between homocysteine (Hcy) levels, gender, methylenetetrahydrofolate reductase (MTHFR) genotypes, and fatty acid (FA) levels. Male gender and the presence of the MTHFR genotype represent a significant risk factor for HHcy in the studied population. A study by Su et al. reports the absence of genetic evidence suggesting a causal association between plasma levels of Hcy, folate, vitamin B12, vitamin B6, and polycystic ovary syndrome (PCOS) in individuals of European ancestry.

Finally, the molecular mechanism responsible for the association between T2DM and atherosclerosis is investigated in a study by Qi et al. who combined identification of differentially expressed genes with bioinformatic enrichment analyses, protein-protein interaction network construction, and core genes identification. The authors also built a transcription factor-mRNA regulatory network and analyzed infiltrating immune cells. Four core genes (IL1B, C1QA, CCR5, and MSR1) that significantly correlate with common immune cells (B cells, CD4+ T cells, regulatory T cells, and M2 macrophages) were identified, together with five transcription factors that regulate their expression (RELA, NFκB1, JUN, TT1, and SPI1). These findings suggest that the interplay between transcription factors, core genes, and immune cells identified in this study may be important for elucidating molecular mechanisms underlying T2DM and atherosclerosis.

Our Research Topic has taken the initial step in assembling novel research findings on potential CMD biomarkers that may stimulate further discussion. Future studies are expected to refine the understanding of the molecular landscape of CMD by further evaluating the prognostic and diagnostic value of molecular biomarkers of CMD reported in this Research Topic.
